# Forgiveness Interventions for Older Adults: A Review

**DOI:** 10.3390/jcm10091866

**Published:** 2021-04-26

**Authors:** Javier López, Maria Inés Serrano, Isabel Giménez, Cristina Noriega

**Affiliations:** Department of Psychology, School of Medicine, Universidad San Pablo-CEU, CEU Universities, 28925 Alcorcón, Madrid, Spain; mariaines.serranofernandez@ceu.es (M.I.S.); i.gimenez3@uspceu.es (I.G.); cristina.noriegagarcia@ceu.es (C.N.)

**Keywords:** aging, forgiving, treatment effectiveness, treatment outcomes

## Abstract

A meta-analysis of the efficacy of forgiveness interventions in older adults was conducted. International databases (Medline, PsycINFO, Scopus, Web of Science) were searched for studies published from 1990 to 2020 that attempted to promote forgiveness in older adults. Most intervention studies are group treatments targeted towards community-dwelling older adults. Participants in these studies are mainly women. The intervention objectives and contents vary widely and often criteria are not well-defined. Participants that received forgiveness interventions reported significantly higher levels of forgiveness than participants that did not receive treatment. Additionally, forgiveness interventions resulted in more changes in depression, stress and anger than no intervention conditions. Forgiveness treatment also enhances positive states (satisfaction with life, subjective happiness, and psychological wellbeing). The reported effects are moderate. The specific treatment model (e.g., Enright’s, Worthington’s) and format (e.g., group-based interventions and individually delivered programs) do not differentially predict better outcomes. In conclusion, future intervention studies should include more male participants and utilize a broader range of follow-up periods. Caution must be exercised because of the limited number of studies developed to date; researchers must be cautious when generalizing the results.

## 1. Introduction

The population in Western societies is getting older. Population aging is one of the greatest policy challenges. Many older adults have become an important source of financial and social support in current families [1]. Older adults are more likely to maintain their physical and mental health by remaining active and preserving their social life. Nevertheless, an increasing number of older adults reach an age at which physical and mental health decline [2,3].

Psychological treatment for older adults is one of the most important factors related to gerontology. Aging presents a challenge in providing adequate mental health services to a population with rapidly increasing longevity. Although older adult patients have many of the psychological needs that other age groups have, they also have some specific needs [4,5]. Older adults have unique mental health care needs. Mental practitioners should demonstrate competence in treating older adult clients (e.g., in cases of mild cognitive impairment or other diseases it is important to know if the older adult is aware of the disease; keeping in mind that depression is not a natural consequence of old age). Nevertheless, most psychotherapy training makes minimal distinctions between middle adulthood and old age clients in the treatment process and even worse, these differences are often associated with ageism [6].

Despite the increased number of older adults, which implies a growing need for psychological interventions, a small proportion of psychologists decide to specialize in working with older clients and their caregivers. There are few intervention studies conducted with older adults and that explicitly take age-specific issues into account. Additionally, understanding age differences in psychological treatments is important for designing treatments that optimize their implementation among older adults and caregivers [7].

Forgiveness is an increasing development area of research within psychology. There is a common understanding of the differences between forgiving, forgetting (losing the memory of the transgression), reconciling (restoring the broken relationship), and other concepts like absolving (declaring free of responsibility the person they accused), condoning (justifying the offense), excusing (alleging reasons or causes to indicate that the transgression was committed in extenuating circumstances)and denying (not recognizing or admitting the offense carried out or the damage caused) [8,9,10]. There is a lack of consensus on the operational definition of forgiveness. Nevertheless, some relevant researchers consider forgiveness a less negative response in general and a more positive one towards the offender [11].

People offer their view of forgiveness considering its unilateral or negotiated nature [12], some offenses can be unforgivable and not everyone can forgive [13].

It is important to highlight the effects of forgiveness on wellbeing. Various studies suggest the physical and mental positive effects of forgiveness [14,15,16], even though it is necessary to also take into account the exceptions or so called “dark side” of forgiveness [17].

Early contributions in the 1970s and 1980s to the forgiveness field involved case studies. Since the 1990s, the use of forgiveness therapy in clinical practice and research has continuously increased [18,19].

Two forgiveness intervention approaches have been used in the majority of forgiveness intervention research to enable clients to forgive a past hurtful event or injustice: these are Enright’s and Worthington’s models. Enright and the Human Development Study Group [20] have developed a 20-step forgiveness model divided into four broad phases (UDWD): Uncovering (presence of negative feelings about the offense), Decision (realizing the need for an alternative resolution in which the client might begin to have a “change of heart” towards the offender), Work (understanding and empathizing with the offending person), and Discovery (finding meaning and universality).

Worthington’s REACH forgiveness intervention model [21] includes five broad steps: Recall (remembering and expressing the painful emotions associated with the offense), Explore (developing the offender’s perspective and motivations, and building empathy), Acknowledge (recognizing times when the victim has received forgiveness from others; giving an altruistic gift of forgiveness), Commit (engaging publicly to forgiveness; making a formal commitment to forgive), and Hold (working to maintain forgiveness over time; holding onto the gains achieved in times of difficulty).

Both models (Enright’s and Worthington’s) define forgiveness and emphasize its potential benefits as well as encourage the development of empathy towards the offender [22]. Although most forgiveness treatments have been conducted in group formats, there are a growing number of studies on individual interventions [19]. Promoting forgiveness in psychotherapy is not merely focused on reducing a lack of forgiveness but also on increasing other positive emotions [23].

Several meta-analyses have analyzed the efficacy of these forgiveness interventions. Results have shown that interventions can effectively promote forgiveness [18,19,22,23,24,25]. Additionally, forgiveness interventions resulted in more changes in depression, anxiety, and hope than no intervention conditions [18,19,22,23,24,25]. It is reasonable to hypothesize that when forgiveness is promoted, anxiety, depression and hopelessness will be mitigated. However, these meta-analyses did not assess if forgiving interventions differed after controlling for patients’ age.

Meta-analyses concerning forgiveness interventions suggest the forgiveness treatment efficacy may be affected by the severity of the offense [18,19,24], level of clinical distress [25], client’s religion [18] and time [18,19,22,23,24]. Furthermore, Konstam et al. suggested that effective forgiveness is associated with age [26]. In addition, a previous meta-analysis pointed out a need for further forgiveness intervention research assessing their effectiveness with older adults [22].

Some general treatments include forgiveness as a part of the treatment skills or outcomes while explicit forgiveness treatments focus on developing forgiveness. Explicit forgiveness treatments appear to promote forgiveness more than general treatments [19]. Forgiveness interventions allow older adults to express their feelings of frustration and anger in a healthy way in which the older adult conveys how they were hurt by the offender. Older adults have usually experienced different harm during their lifetime. Harm results from everyday acts of violence: bullying in infancy, partner violence in adulthood and abuse in old age [22]. Hebl and Enright pioneered the first formal forgiveness psychotherapy study, and they implemented the first forgiveness intervention with female older adults. This pioneering study selected participants older than 65 years of age because of the unique interpersonal struggles the elderly face. Nevertheless, a more systematic assessment of older adults’ intervention studies is needed [27].

Although forgiveness interventions appear effective for promoting forgiveness and mental health, questions about how aging affects the forgiveness process remain unaddressed. Specifically, about what factors are more likely to facilitate an older participant’s response to treatment. This article offers a systematic review of studies on the effectiveness of different forgiveness intervention programs focused on older adults. To our knowledge, this is the first study that reviews forgiveness interventions with this age group.

## 2. Materials and Methods

This review was performed following the Preferred Reporting Items for Systematic Review (PRISMA) guidelines [28].

### 2.1. Search Strategy, Eligibility Criteria and Study Selection

A systematic review of the literature was carried out from 1990 to 2020, since it is precisely in the 90s when systematic studies of forgiveness interventions with older people began [18,19]. We searched the following areas to locate studies for inclusion: (a) computerized search of the PsycINFO, MEDLINE, Web of Science (WOS), and Scopus databases using keywords: “forgiv*”, “elderly”, “aged”, “elder”, “older”, “intervention”, “treatment”, “therapy” and (b) manual search of references listed in all located studies. Studies were included if they (a) involved older adult clients (participants aged 55 and over or studies with a mean or median age of ≥60 years) who received a forgiveness intervention; (b) offered the treatment in-person by a trained facilitator with sufficient detail to be replicated, (c) were written in English or Spanish, and (d) were completed from 1990 to 2020. Studies were excluded if they were self-help rather than therapist-led (e.g., online resource or book).

Relevant terms were integrated with Boolean conjunction (OR/AND) for search based on three search levels: (i) elderly [Abstract] OR aged [Abstract] OR older [Abstract] OR elder [Abstract] OR geriatric [Abstract] AND (ii) intervention [Abstract] OR treatment [Abstract] OR therapy [Abstract] AND (iii) “forgiv*” [Abstract].

**Figure 1:**
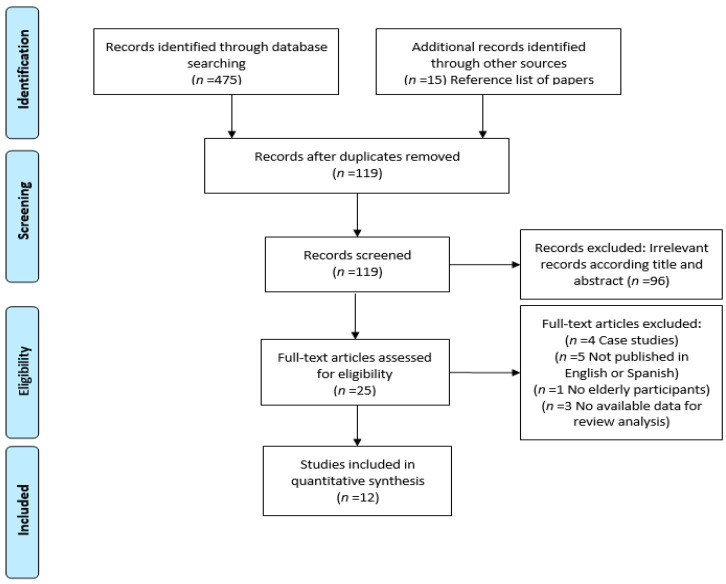
PRISMA Flow Diagram.

In Figure 1, we display the numbers of found, eligible, and ineligible studies. The first author (J.L.) took overall responsibility in designing, conducting and reporting the review. The second author (M.I.S.) advised whether studies met the inclusion criteria. The third author (I.G.) rated the risk of bias. The second, the third and the fourth authors (M.I.S., I.G., C.N.) also checked the review to ensure that all data extracted and inputted for analysis were correct.

### 2.2. Data Items and Collection Process

Twelve studies (six studies were conducted in the US, two in Switzerland, Korea, and Spain) were selected. Of these, three [29,30,31] did not employ a comparison group (e.g., a control or alternative treatment group). The study characteristics of the selected interventions are summarized in Table 1 and are presented alphabetically. Data items in the extracted table include references (authors, year of publication, and country); study characteristics (final sample and attrition rate) and participant characteristics (age mean/age range, setting, and percentage of females); intervention model (Enright’s, Worthington’s or some Other) and control or comparison group characteristics; intervention protocol (the type of intervention and intervention duration); intervention characteristics (mode, number of sessions, dosage-hours, pre, post and follow-up assessment) and outcomes.

## 3. Results

### 3.1. Participants

Most studies use convenient samples (volunteers, advertisements, senior centers, etc.). Taken together, the studies reported findings from 451 participants. Final sample sizes ranged from 19 [30] up to 78 [32]. The number of participants can be considered small (n < 30) in four studies.

Most of these interventions focus on healthy older adults to use adaptive forgiving skills (e.g., recalling the specific harm and emotions associated with the transgression; and understanding and empathizing with the offending person), except for one study in which participants from one study had a confirmed diagnosis of terminal cancer with 6 months or less to live. Most of the studies also focus on older people residing in the community (91.6%). Others use samples of institutionalized older adults in nursing homes.

Most studies also reported an average age between 70 and 80 years. One of the studies was designed as an intervention for participants over 65 years of age and reported an average age for the entire sample of 74.5 years [27]. Two studies included participants aged 57 years and older, but did not state their mean age [30,37].

Female percentages in the selected studies ranged from 51% to 100%. Only three studies included less than 80% of female patients. Even though participants usually have a medium level of education, the percentage of participants with university studies is high (in two studies it is between 34% and 50%).

The types of transgressions reported were mostly emotional and/or verbal abuse (28–60%), bullying, harassment or lack of appreciation (28%), and being emotionally neglected (15%) [27,32]. Participants also reported that the transgressions had been mostly committed by a romantic partner (27–30%) and/or a family member (28–39%) [30,32,33]. Regarding time since the transgression had occurred, participants reported that they experienced the transgressions between 10 and 20 years ago (14–18%), and more than 20 years ago (15–29%) [27,32,33].

Another source of heterogeneity was participant attrition (dropouts). This ranged from 2% of participants to up to one-fifth of the sample [38]. Dropout rates did not vary by model of intervention and the reasons for withdrawal were diverse. The reasons given for dropping out included not being able to forgive yet, being too busy, feeling uncomfortable with sharing intimate feelings in a group setting, change of residence, physical illness or injury, and death.

### 3.2. Methodology

There were various recruitment strategies implemented. Recruitment information was sent to health care professionals within geriatric clinics, senior centers (and professionals involved in the university in the departments of psychiatry, social work, and geriatrics). Word of mouth was used to recruit participants. Flyers describing the treatment were also sent to the community (e.g., libraries, hospitals, churches, synagogues, local newspapers, radios and agencies serving older adults) and at events attended by older adults [29,30,34,39]. Letters of invitation were also sent to a specific Christian church community in one study [27].

Participants’ recruitment was mainly achieved through professional referral and advertisement, except for two studies that also recruited from nursing homes [31,36], and another one that invited clients from senior citizens’ day centers [38].

The 12 research reports included data on 13 interventions designed to promote forgiveness. Nevertheless, five of these treatment groups were in studies that did not include a control group. Most of the studies compared a forgiveness intervention to a non-specific treatment (*n* = 3). The remaining studies used a waiting-list group (*n* = 2), a placebo group (*n* = 2) or a usual care group (*n* = 1).

There was a variation of forgiveness measures used across the different interventions. The most popular measures of forgiveness were the Enright Forgiveness Inventory (EFI) [40] and the Transgression-Related Interpersonal Motivations Inventory (TRIM) [41]. The EFI is a 60-item forgiveness self-report questionnaire. The victim of an offense is asked to consider the offender and rate that person based on their current positive and negative emotions, cognitions, and behaviors towards the offender. Items are rated on 5-point Likert scales. Scores range from 60 to 300. Higher scores indicate more forgiveness. Six domains (10 items each) are assessed: presence of positive emotions (e.g., [Regarding the person offender] I feel warm), presence of positive cognitions (e.g., I think he or she [offender] is worthy of respect), presence of positive behaviors (e.g., [Regarding the person offender] I do or would show friendship), presence of negative emotions toward the offender (e.g., I feel repulsed by the offender), presence of negative cognitions (e.g., I think he or she is wretched), and presence of negative behaviors (e.g., I would avoid the offender). To avoid response set bias, the word forgiveness is not used in any of the 60 items.

The TRIM is also a commonly used forgiveness measure. It is an 18-item questionnaire. Items are rated on five-point Likert scales. Scores range from 18 to 90. Higher scores indicate more unforgiving motivations (i.e., less forgiveness). This inventory assessed the reasons for not forgiving (motivation for revenge and avoidance) and reasons for forgiveness (motivation for benevolence). The avoidance subscale uses seven items to assess motivation to avoid the aggressor (for example: “I live as if he/she does not exist, as if he/she is not around”). The revenge subscale, with five items, assesses motivation to seek revenge (for example: “I’ll make him/her pay “). Finally, the benevolence subscale consists of six items and assesses the motivation for benevolence (for example: “Even though their behavior hurts me, I am benevolent towards him/her”).

Several well-known measures of negative affect were used. Depression was commonly measured using the Beck Depression Inventory or the Geriatric Depression Scale. Anxiety was usually measured using the State-Trait Anxiety Inventory. Feelings of stress were often measured by the Perceived Stress Scale and anger was usually measured using the State-Trait Anger Expression Inventory.

Positive affect is also assessed. One measure of satisfaction with life was used, the Diener Satisfaction with Life Scale. As a measure of happiness, the Lyubomirsky Subjective Happiness Scale was used.

Of all the studies, six used follow-up assessments at two weeks [32], 1 month [33,35], 3 months [29] and 4 months [30,38].

Data analysis usually includes repeated measure analysis of variance and t-test. It should be noted that, despite numerous dropouts, no investigation used intention-to-treat in analysis.

### 3.3. Intervention Characteristics

Three studies reported forgiveness interventions using Worthington’s model. Two studies used Enright’s model, including learning-oriented and action-oriented treatments. Seven studies used other forgiveness models, of which four follow a positive-psychology framework.

The objectives of the forgiveness programs are diverse and sometimes unspecific. Most intend to promote forgiveness in older adults and, in turn, improve their emotional state.

The content and development of the interventions were not always sufficiently specified. Those that appear most frequently are psychotherapeutic programs (seven studies), in a number very similar to psychoeducational programs (five studies). Despite the effort made to specify the type of intervention, it is difficult to determine if an intervention is strictly psychoeducational or if it is psychotherapeutic. In general, there is an evolution in the contents of the programs: from the presentation of specific forgiveness programs based on Enright or Worthington models in the older ones, to the most recent ones offering specific forgiveness strategies and tools within broader interventions of positive psychology. For their application, various techniques are used (group discussion, readings, audiovisual materials, etc.), without homogeneity between the different forgiveness interventions.

Twelve were conducted through group sessions and only one as an individual intervention. The duration of forgiveness treatments varied greatly, and had an average duration of 7 weeks (range 2–12) weeks), which was similar to forgiveness intervention durations reported for patients of all ages in a previous meta-analysis [22]. Groups were led and supervised by a trained facilitator who was usually a social worker (four studies) or a psychologist (three studies).

In only one study, the intervener conducted sessions at the participants’ homes [35]. Additionally, one group intervention used occasional phone contacts [29].

Each session varied greatly (50 to 150 min). However, in most cases, especially in psychotherapeutic treatments, the sessions lasted approximately one hour.

Only four studies assessed patients’ implementation. Average attendance to sessions was 85–90% in forgiveness intervention and 81% in control group intervention [27,30,37,39]. Data seem to indicate good adherence to treatment. In only one study, an observer attended the interventions weekly to monitor participants’ engagement and topic discussions. This study also evaluated the intervention completion, completion of homework assignments and verbal encouragement during assignment sharing and discussions. Moreover, in this study some participants were interviewed at the end of the intervention about program implementation [39].

Finally, therapists’ fidelity in implementing each program was confirmed. To ensure that therapists were not biased in sessions, these were manualized [27,32,35,37], audio-taped and rated by experienced professionals [27,35].

### 3.4. Results

As only two studies investigated physical health outcomes, this dependent variable was dropped [30,37]. The empirical evidence supporting forgiveness interventions showed effect sizes, when these data are offered, from 0.008 to 1.87. Nevertheless, only two studies showed an effect size over 0.8 [31,35]. The magnitude of effect sizes includes 0.2 as small, 0.5 as moderate, and 0.8 as large [42]. Consequently, across forgiveness and mental health variables, the effect of this set of interventions can be considered small to medium-sized effects for decreases and increases in the outcome variables. The 0.5 effect size on forgiveness can be considered in terms of the average person in the intervention group doing as well as or better than 69% of the control group. Therefore, forgiveness and mental health variables can be affected by forgiveness intervention.

Studies have indicated that forgiveness interventions with older adults can effectively promote others’ forgiveness [27,29,30,32,33]; but not self-forgiveness among institutionalized older adults [36]. Additionally, forgiveness interventions reduced depression [29,30,31,32,33,34,35,38,39,40], anxiety [27,38], psychological distress [33,39] and anger [33,35]. Forgiveness intervention also reduced rumination in older adults, a cognitive response style characterized by repetitive thinking which relates to depression [29,33]. On the other hand, some studies found no significant changes in anxiety [29,30] while others observed changes in death anxiety [36].

Forgiveness interventions with older adults are effective not only in “attenuating the negative,” but also in enhancing positive states as indicated by increases in satisfaction with life [31,33,36,38,39], subjective happiness [31,34,38,39], and psychological wellbeing [29,31].

Learning-oriented and action-oriented forgiveness interventions were both effective in decreasing thoughts of revenge, transgression-related thoughts and feelings, negative affect, and psychological distress as well as providing increases in life satisfaction. Nevertheless, there is only one study focused on these differences. Therefore, this result needs replication in future studies.

Regarding the effects of the different types of interventions, the data indicate that psychotherapy achieves better results than psychoeducation, especially on intervention effect size.

### 3.5. Study Quality Assessment

Study quality of all the selected studies is summarized in Table 2. The selected studies demonstrated fair-to-medium study quality. It was noted that allocation concealment was only used in one of the selected studies. Studies did not use intention-to-treat analysis for missing data.

Overall, the graph shows that approximately 58% of the studies posed a low risk of bias regarding detailing the method of randomization. In 75% of the studies, it was unclear if a method of allocation concealment was used. Approximately 90% of included studies posed a high risk of bias concerning single blinding. In about 25% of the studies, the outcome assessor was not blinded and the measures were administered during a face-to-face session. Finally, 83% of studies were rated as low risk on levels of attrition bias (dropout rates were less than 30% or differed by less than 10% between the experimental and control groups dropout rates).

## 4. Discussion and Conclusions

As the first systematic review synthesizes the evidence of the effects of forgiveness intervention on older adults, we found that forgiveness treatments may have positive effects on older adults, with no occurrence of significant adverse events. This evidence suggests that there is a possibility for using these interventions as an alternative and/or augmentation approach to conventional treatments for older adults.

Despite the increased number of geriatric populations that implies a growing need for psychological treatments, only a few psychologists decide to specialize in psychotherapy with older adult patients. Understanding age differences in psychological treatments is important in order to design mental health interventions to optimize their implementation among older adult patients [43,44].

In addition, the empirical study of forgiveness interventions aimed at older adults began in the 1990s, and it is still not possible to clearly establish the effectiveness of this type of intervention. Treatments have not been homogeneous and have admitted a variety of content and formats, so it seems appropriate to carry out a detailed analysis of what has been done in order to optimize future forgiveness interventions.

Forgiveness interventions are not only effective in decreasing negative states and increasing positive states with younger age groups, but also with older adults. Mental health practitioners must be aware of unresolved transgressions in older clients and should carefully make them a subject of treatment.

Overall, our results suggest at least the possibility that older adults might benefit from forgiveness interventions, including those provided in a group setting. On the one hand, group members could empathize with each other’s harm, help to reframe thoughts about the painful person, suggest new behaviors, and offer forgiving models [30]. On the other hand, in their meta-analyses individual interventions were more effective than group interventions [21,22]. Nevertheless, only one forgiveness intervention with older adults was delivered in one-on-one sessions, and the others were group treatments emphasizing social support and participant interaction. Furthermore, none of the studies compared the efficacy of group versus individual forgiveness treatment formats.

Although the duration of the forgiveness interventions varied across the included studies, it is possible that in most studies, older adult participants had attained the minimal duration needed to obtain the psychological benefits. Findings from past meta-analysis on typical patterns of forgiveness in all age patients [18,19,20,21,22] found that treatment dosage was an important predictor of forgiveness. With more treatment time, patients are generally able to develop more forgiveness. Research has suggested that forgiving takes time. Nevertheless, a previous meta-analysis [18] indicated the optimum duration for a forgiveness group intervention is six hours. Although different populations and formats may require longer intervention durations, only one study selected in our review with older adults spent less than six hours [35]. One of the strongest effects of the intervention was found for perceived painfulness of the transgression, particularly in the post-treatment phase [32]. This might indicate that changes in the process of forgiveness are rather slow and can take a long time. Program length matters in designing a forgiveness treatment. Results suggested that a higher number of sessions conducted throughout a longer length of time are linked to better outcome and success. Our findings suggest that treatment duration might be a key variable in forgiveness treatment. Forgiveness in older adults seemed to take time.

The inability to forgive is often associated with negative emotions such as depression, stress, anxiety and anger, all of which can detract from quality of life, satisfaction with life, hope and subjective happiness [22]. Nevertheless, if the intervention did not increase forgiveness, the conclusion that other outcomes (e.g., life satisfaction, death anxiety) were positively affected may not be appropriate. Forgiveness therapy was more effective at helping older adult participants achieve forgiveness, improve positive emotions and reduce depression and other negative emotions for late life compared to the waiting list, non-specific treatment conditions, and usual care (i.e., daily activities at their nursing home). However, the effect was lost when other active conditions were employed in comparison, such as a discussion group [27]. Caution is required when interpreting these results, as there were relatively few studies involved and they were likely to have been underpowered to detect the superiority of one effective forgiveness treatment over another. Furthermore, follow-up assessment varied greatly and in none of the studies did it reach six months or more. Moreover, some relevant variables such as quality of life could be assessed in future studies [45].

Full forgiveness treatments (interventions that incorporate all components of a treatment model) were, in fact, more effective than partial treatments (dismantled interventions that used only certain components of a model) [19]. Nevertheless, many studies selected in this review only apply some forgiveness components or forgiveness is included in a broader intervention. In any case, it seems correct to assume that the intervention must be carried out by well-trained facilitators, who can offer specific skills to improve levels of forgiveness.

Allemand et al. [32] found that almost half of the sample had recovered from a serious transgression that occurred 10–20 years ago. Moreover, forgiveness therapy had good results with older adults even when some of the people that had caused the harm were deceased [30]. Forgiveness interventions improve the affective condition of older adults related to transgressions that happened a long time ago and are still unresolved. Therefore, forgiveness interventions with older adults with past harm are highly recommended.

Women are, without any doubt, the group that has received the most attention. A significant part of the studies carried out is focused on female older adults. This preponderance is based on the consideration that older women are in a situation of greater vulnerability, which causes them to develop greater emotional problems and a greater need for forgiveness. The overrepresentation of women in the samples in relation to the number of men is noteworthy, which also seems to indicate that older women are more likely to participate in forgiveness interventions.

Older adults have traditionally been presented as a group that is difficult to access. Many are not interested in participating in the different interventions and others, even if they are interested, have serious difficulties that prevent them from getting involved. Moreover, in general, they show little initiative in the use of resources. The sample size of the studies considered here (generally medium or low) corroborates this statement. Investigations of forgiveness interventions with older adults with larger sample sizes would be desirable.

Although the forgiveness interventions improve older adults’ emotional levels, the participants do not necessarily have to be emotionally affected (e.g., depression, stress, anger). Few studies focus their interventions exclusively on emotionally affected older adults, which can cause a floor effect to occur that makes it difficult to find significant improvements. It is important to keep in mind that it is more difficult for older adults highly affected on an emotional level to assimilate new concepts and information.

The medium quality treatments in this systematic review showed that forgiveness intervention is a treatment that is safe and effective enough for older adults. Nevertheless, some limitations should be acknowledged, involving the inclusion criteria we employed, the heterogeneity of the available clinical treatments, and the analyses performed. Firstly, although we established wide inclusion criteria, only a relatively small sample of studies was obtained. Running fewer participants than needed may pose a substantial risk of reaching incorrect conclusions [46]. The indexes of study quality show that there are only a few studies that used random selection and double-blind design. Therefore, the results should be interpreted carefully. Nevertheless, the only forgiveness studies included were those with sufficient detail to be replicated, and that were offered as interventions in-person by a trained facilitator. In this way, we aimed to increase the quality of the included trials and hence the reliability of our findings.

Secondly, allocation concealment in half of the selected trials was absent in this systematic review. Such inadequate concealment of allocation was associated with exaggerated estimates of forgiveness treatment benefits for older adults.

Another limitation concerns the therapeutic heterogeneity between studies (Enright, Worthington and some other forgiveness models) that might reduce the generalizability of observed treatment effects. However, response definition was reasonably consistent within the forgiveness model. A previous meta-analysis [23] of forgiveness interventions for patients of all ages observed that when dosage (hours) and modality (individual or group) were both controlled, the treatment model (Enright or Worthington model) was not a significant predictor of study effect size. Both models did not differ in efficacy.

Fourth, forgiveness was not offered as monotherapy in half the number of studies, but as adjunctive treatments to existing interventions (positive psychological therapy or body–mind–spirit therapy). It may be difficult to conclude whether the positive outcomes were attributed to the forgiveness intervention alone, a synergetic intervention effect, or to the conventional treatment received by the patients. Nevertheless, results from our overall analysis provide support for forgiveness intervention as an adjunctive treatment or as a monotherapy for older adults.

Fifth, it is also a limitation that cognitive functioning was not assessed in the included studies. Unmeasured cognitive impairment could have interfered not only with the report of forgiveness levels but also with some emotional symptoms themselves, as mild cognitive impairment is associated with increased anxiety and depression [47]. In half of the studies reviewed, one of the exclusion criteria was moderate or severe cognitive impairment [29,31,34,35,38,39]. Therefore, samples usually consisted of healthy and highly motivated older adults. These resources may enhance the capacity and the willingness to forgive. Participants were mostly cognitively healthy and the studies involved in this review did not analyze the differences in terms of treatment efficacy between the older individuals with and without signs of cognitive deterioration or with mild cognitive impairment. Finding a way of doing this is a challenge for future forgiveness research.

Furthermore, previous research suggests that older adults with dementia or mild cognitive impairment have limited opportunities for forgiveness treatments. Many times, they are excluded from treatments. Remembering the offense is a keystone of forgiveness treatments. Interventions encourage participants to remember the hurt (and the associated behaviors, thoughts, and feelings) as fully as possible [21] or they encourage a deep exploration of the consequences of the hurt [20]. Forgiveness treatments could be adapted for use with people with cognitive impairment and may offer an alternative treatment. Modifications from usual forgiveness interventions could include repeated instructions, more in-session practice, reminder cues, and simplified sessions.

Although a respectable percentage of studies included follow-up assessments of the intervention conditions, fewer reported follow-up data for control group patients who often entered the treatment phase following the post-assessment. Therefore, this limits our confidence in our results about how people who are not in treatment or who are waiting for treatment change in terms of forgiveness over a longer time. This limitation is also included in a previous meta-analysis about forgiveness intervention for the global population [19].

Usually, in the literature about aging older adults, participants are over 65 years old. Current reviews include participants over 55 years old or studies with a mean or median age of ≥60 years of age because only three studies included merely participants over 65 years old. Nevertheless, nine studies had a mean age of ≥70 years.

Finally, there is an overrepresentation of female participants. The difficulty of recruiting men for forgiveness treatment research is a common problem that needs more attention in future studies. Nevertheless, females in this age group have significant psychological pain characterized by guilt, abandonment, loneliness and poverty. People in this group can lose former status [27]. Certainly, male older adults are a group for which forgiveness intervention is suitable, but the female population represents a sample with diverse difficulties, making it especially relevant for forgiveness treatment research.

Despite such limitations, the present review does provide evidence that a structured forgiveness treatment may be helpful for older adults. The strengths of our review include the comprehensive literature search conducted, focused on older adults, and the inclusion of Enright, Worthington and other psychotherapeutic models. Furthermore, we have included only studies that offered in-person treatments by a trained facilitator. Overall, this has allowed us to obtain a more precise estimate of current forgiveness intervention efficacy in older cohorts. Given the fact that forgiveness treatments are safe and easily accessible, clinicians may consider recommending forgiveness interventions for older adult patients.

The recent COVID-19 pandemic has shown the older adults’ strengths. It has been suggested that the COVID-19 outbreak has a limited impact on older adults’ psychological wellbeing [8]. Nevertheless, forgiveness treatments are helpful for many older adults and many kinds of harm. One promising tool for mental health practitioners in diverse health care settings is forgiveness therapy with older adults.

## Figures and Tables

**Table 1:** Characteristics of forgiveness intervention studies in aged care clients (1990–2020).

Author, Country	*N* (Attrition Rate)	Age (Mean Years) Setting Gender	Intervention Model and Control Group	Therapeutic Approach Treatment Length	Mode, Sessions, Dosage (Hours) Assessments	Results
Allemand et al., 2013 [32] Switzerland	*N* = 78 (3.7%)	70.1 years (range 50–90 years) – 75.6% women	EG: Worthington Model CG: Waiting list	Psychoeducational ½ month	Group 2 sessions (7 h) Pre, post, ½-month follow-up	The intervention reduced the levels of perceived actual transgression painfulness, transgression-related emotions and cognitions, and negative affect
Allemand and Flückiger, 2020 [33] Switzerland	*N* = 73 (15%)	68.8 years (range 57–82 years) – 84% women	EG1: Worthington Model (learning-oriented, *n* = 39) EG2: Worthington Model (action-oriented, *n* = 34)	Psychoeducational ¾ month	Group 3 sessions (10.5 h) Pre, post, 1-month follow-up	Both interventions resulted in decreases in revenge, transgression-related thoughts and feelings, negative affect, and psychological distress as well as increases in life satisfaction
Foulk et al., 2017 [29] USA	*N* = 40 (11.1%)	71.1 years (range 57–90 years) Community-dwelling 76% women	EG: Other Model (Luskin, Mindfulness-based forgiveness)	Psychotherapeutic 2 months	Group 8 sessions (20 h) Pre, post, 3-month follow-up	The intervention significantly increases forgiveness, mindfulness/self-compassion, and mental health
Greenawalt et al., 2019 [34] USA	*N* = 52 (---%)	71.2 years (50 years of age and older) Senior centers clients 88.5% women	EG: Other Model (The Art of Happiness. Forgiveness is one of the 8 topics included) CG: Nonspecific Treatment	Psychoeducational 2 months	Group 8 sessions (12 h) Pre, post	The intervention reduces perceived stress, and tiredness. It also increases the calm
Hansen et al., 2009 [35] USA	*N* = 20 (---%)	73 years (range 62–84 years) Older adults terminally Ill Cancer patients with 6 months or less to live 90% women	EG: Enright Model CG: Waiting list	Psychotherapeutic 1 month	Individual 4 sessions (4 h) Pre, post, 1-month follow-up	The intervention increases forgiveness, hope, quality of life, and it also reduces anger
Hebl and Enright, 1993 [27] USA	*N* = 24 (7.7%)	74.5 years (>65 years) Members of a Christian community 100% women	EG: Enright Model CG: Placebo. Discussion topics (e.g., homeless, morals of the young, nursing home care, influence of senior citizens on society, societal patterns of drug abuse, attitudes toward aging, and family conflicts) avoiding specifying the topic of forgiveness	Psychotherapeutic 2 moths	Group 8 sessions (8 h) Pre, post	Participants in the forgiveness condition, relative to the control group, showed significantly higher levels of forgiveness. In both groups, depression and anxiety decreased significantly.
Ingersoll-Dayton et al., 2008 [30] USA	*N* = 19 (5%)	- (aged between 57 and 82 years) - 80% women	EG: Enright Model	Psychotherapeutic 2 moths	Group 8 sessions (16 h) Pre, post, 4-month follow-up	Participants experienced long-term improvement with respect to forgiveness and depression and short-term improvement of physical health.
Jo and An, 2018 [36] Korea	*N* = 47 (2%)	EG= 77.79 years; CG = 80.16 years (range 69–91 years) Nursing homes 51% women	EG: Other Model (Reminiscence Program on Self-forgiveness) CG: Daily activities at their nursing home	Psychotherapeutic 2 moths	Group 8 sessions (6.66 h) Pre, post	The intervention increases life satisfaction and reduces death anxiety levels
Lee et al., 2018 [37] Korea	*N* = 70 (11.42%)	– Community-dwelling –	EG: Other Model (Body–Mind–Spirit Program. Forgiveness is one of the 12 topics included) CG: Nonspecific Treatment	Psychoeducational 3 moths	Group 12 sessions (hours) Pre, post	In terms of physical health, the program was effective in enhancing overall rating of health, activity levels, sleeping habits, knowledge on nutrition, and attitude toward sexuality. Participants reported significant improvements in levels of peace of mind, life satisfaction, and optimism about the future.
Ortega et al., 2015 [31] Spain	*N* = 26 (13.33%)	81.69 years (range 69–91 years) Institutionalized 63% women	EG: Other Model (Memories, gratitude, humor and forgiveness)	Psychotherapeutic 2 and ¾ moths	Group 11 sessions (11 h) Pre, post	Intervention decreased depression as well as increased specific memories, life satisfaction, purpose in life, gratitude and subjective happiness.
Ramírez et al., 2014 [38] Spain	*N* = 46 (17.86%)	71.18 years (range 60–93 years) Senior Citizens’ Day Centre 63% women	EG: Other Model (Memories, gratitude and forgiveness) CG: Placebo Positive Psychology group	Psychotherapeutic 2 and ¼ months	Group 9 sessions (13.5 h) Pre, post, 4-month follow-up	Intervention decreased state anxiety and depression as well as increased specific memories, life satisfaction and subjective happiness
Turner et al., 2017 [39] USA	*N* = 34 (-%)	70.91 years (50 years of age and older) Senior centers clients 85.2% women	EG: Other Mod-el (The Art of Happiness. Forgiveness is one of the 8 topics included) CG: Nonspecific Treatment	Psychoeducational 2 months	Group 8 sessions (12 h) Pre, post	The intervention increased participant’s subjective happiness, satisfaction with life and overall mindfulness Program also reduced stress levels, depression scores, tension and tiredness

CG = Control Group; EG = Experimental Group.

**Table 2:** Risk of bias.

Author	Selection Bias	Allocation Concealment	Performance Bias	Detection Bias	Attrition Bias
	Random Sequence Generation		Single Blind	Blinding of Outcome Assessor	Missing Data
Allemand et al., 2013 [32]	+	?	+	?	+
Allemand and Flückiger, 2020 [33]	+	?	?	?	+
Foulk et al., 2017 [29]	?	?	?	?	+
Greenawalt et al., 2019 [34]	−	?	?	?	?
Hansen et al., 2009 [35]	+	?		?	+
Hebl and Enright, 1993 [27]	+				+
Ingersoll-Dayton et al., 2008 [30]	−	?	?	?	+
Jo and An, 2018 [36]	−	−	?	?	+
Lee et al., 2018 [37]	−	−	?	?	+
Ortega et al., 2015 [31]	−	?	−	?	+
Ramírez et al., 2014 [38]	+	?	?	−	−
Turner et al., 2017 [39]	?	?	−	?	+

+, low risk of bias; −, high risk of bias; ?, unclear risk of bias.

## Data Availability

Not applicable.
